# Unveiling the Complexity in the Management and Outcomes of Traumatic Versus Non-Traumatic Bile Leaks—A Comparative Analysis

**DOI:** 10.1007/s10620-025-09550-6

**Published:** 2025-11-22

**Authors:** Atul Lodh, Dalton A. Norwood, John A. Cooper, Ramzi Mulki, Sergio A. Sánchez-Luna, Ali M. Ahmed, Kondal R. Kyanam Kabir Baig, Shajan Peter

**Affiliations:** 1 Division of General Internal Medicine and Population Science, University of Alabama at Birmingham, 1720 2Nd Avenue South, BDB 327, Birmingham, AL 35294 USA; 2 Division of Gastroenterology and Hepatology, University of Alabama at Birmingham, 1808 7Th Avenue South, BDB 391, Birmingham, AL 35294 USA; 3 Division of Gastroenterology and Hepatology, Basil Hirshowitz Center of Advanced Endoscopy, University of Alabama at Birmingham, Birmingham, AL 35294 USA

**Keywords:** Endoscopic retrograde cholangiopancreatography, Bile duct injuries, Bile leak, Traumatic injuries

## Abstract

**Background and Aims:**

Endoscopic retrograde cholangiopancreatography (ERCP) is a crucial diagnostic and therapeutic modality for managing bile leaks, particularly in the context of traumatic etiologies. Here, we aimed to contribute to this body of knowledge by providing a comprehensive analysis of ERCP outcomes in patients with confirmed bile leaks, including both traumatic and non-traumatic etiologies.

**Methods:**

This retrospective study investigated ERCP outcomes in patients with confirmed bile leaks from 2017 to 2023. Patients were categorized into non-traumatic (N = 124) and traumatic (N = 47) groups, collecting variables encompassing demographics, clinical parameters, and intervention details. The comparisons between groups utilized Chi-square for categorical variables.

**Results:**

This study analyzed 188 bile duct leak cases, comparing 141 non-traumatic and 47 traumatic injuries. Logistic regression identified younger age (< 40 years; aOR:19.75, p < 0.001), male sex (aOR:6.45, p = 0.001), and Black race (aOR:3.41, p = 0.019) as significant predictors of traumatic injury. Hospital stay was significantly longer in traumatic cases (21.3 vs 12.4 days; p = 0.002), reflecting greater injury severity and complexity, including a higher need for interventional radiology (59.6% vs 33.3%; p = 0.001). Despite these differences, clinical outcomes, including stent success and complication rates, were comparable between groups.

**Conclusion:**

Our findings underscored a higher frequency of non-endoscopic involvement in traumatic injuries, indicating the complexity of these cases. Despite these differences, the study revealed that technical success, clinical success, mortality, and complications do not significantly differ between traumatic and non-traumatic groups, emphasizing the need for tailored management strategies outside of ERCP to address the distinct clinical characteristics of traumatic bile leaks.

**Supplementary Information:**

The online version contains supplementary material available at 10.1007/s10620-025-09550-6.

## Introduction

Endoscopic retrograde cholangiopancreatography (ERCP) is a pivotal tool in the management of bile leaks [1]. Bile leaks can result from either traumatic injuries (penetrating or blunt trauma) or as a post-surgical complication, such as after cholecystectomies [2]. Bile leaks are also common after liver transplantation, hepatic trauma, and partial hepatectomy [1]. The incidence of bile leaks following hepatobiliary trauma ranges from 0.5 to 21% [2]. Compilation from the literature suggests the incidence of bile leaks secondary to non-traumatic etiologies ranges between 2 and 25% [1, 3, 4]. Traumatic bile leaks can lead to significant morbidity and mortality as well as lengthier hospital stays [5]. Post-surgical bile leaks as a non-traumatic etiology are one of the most serious complications and can lead to sepsis, liver failure, and possibly even death [6]. Though advancements in endoscopic techniques continue to grow, ERCP remains at the forefront of managing bile duct leaks, offering patients a less invasive yet highly effective treatment modality [1]. Outside of trauma patients that have obvious indications for open exploratory laparotomy and damage control surgery (e.g. hemodynamic instability, multiple effected organs), ERCP allows for both diagnostic and therapeutic intervention in these cases, allowing for a comprehensive approach to addressing these bile leaks [5].

The effectiveness of ERCP for the treatment of both traumatic and non-traumatic bile leaks, including penetrating injuries, blunt traumas, and post-operative complications, has been well established. Management of bile duct leaks non-operatively have shown success rates of up to 90% [2]. Multiple cases series of a small subset of patients undergoing ERCP for management of bile leaks resulting from trauma and blunt abdominal injury showed that bile leak resolution was seen in almost all patients that had ERCP with endobiliary stent placement done [2, 7, 9]. A retrospective study examining the effectiveness of ERCP management for postoperative, non-iatrogenic bile duct leaks also displayed success for all patients included [8]. In addition, retrospective studies of patients who underwent bile leaks after liver injury demonstrated that a majority of patients who underwent ERCP had successful bile leak control [10, 11]. However, recent literature providing a comparative analysis of patients requiring ERCP for the management of bile duct leaks of various traumatic and non-traumatic causes is scant.

Here, we aim to contribute to the literature by providing a comprehensive examination comparing the differences in management complexity and outcomes of patients with traumatic versus non-traumatic bile leaks. Specifically, we compared demographics, clinical parameters, and intervention details in patients who underwent ERCP for traumatic versus non-traumatic bile leaks. In doing so, we hope to understand the differences in therapeutic interventions needed to manage traumatic and non-traumatic bile leaks and shed further light on the outcomes for these patients.

## Methods

### Patient Population

We performed a retrospective review of patients who underwent ERCP for suspected biliary leak between May 22, 2017 and October 9, 2023 at the University of Alabama at Birmingham, an academic tertiary care center. The study population was identified by querying of the institution’s Provation database using the following diagnoses: established bile leak, suspected bile leak, follow-up of bile leak, biliary leak on CT, pancreatic duct leak. These corresponded to the following Current Procedural Terminology (CPT) codes: 51.36, 51.37, 51.39, 51.71, 51.72, 51.84, 52.98, and 51.79 (see Supplement Table 1 for CPT descriptions). Dataset depuration was performed in the following stages: (1) removing patients who did not undergo ERCP, (2) excluding cases in which no leak was identified, (3) removing duplicate cases. Patients were categorized based on indication for ERCP as either non-traumatic or traumatic. The suspicion for bile leak that resulted in plans for endoscopic evaluation with ERCP was established based on imaging results.

**Table 1:** Table of General Characteristics of the population by mechanism of injury (Non-traumatic vs traumatic)

	Non-Traumatic N = 141	Traumatic Injury N = 47	Total N = 188	p-value
Age [years], mean (SD)	54.4 (17.7)	30.0 (13.4)	48.3 (19.8)	< 0.001
Age Category				< 0.001
< 18	0 (0.0%)	5 (10.6%)	5 (2.7%)	
18–25	9 (6.4%)	15 (31.9%)	24 (12.8%)	
26–40	30 (21.3%)	19 (40.4%)	49 (26.1%)	
40–60	44 (31.2%)	6 (12.8%)	50 (26.6%)	
> 61	58 (41.1%)	2 (4.3%)	60 (31.9%)	
Gender				< 0.001
Female	75 (53.2%)	8 (17.0%)	83 (44.1%)	
Male	66 (46.8%)	39 (83.0%)	105 (55.9%)	
Race				< 0.001
White	108 (76.6%)	15 (31.9%)	123 (65.4%)	
Black	28 (19.9%)	32 (68.1%)	60 (31.9%)	
Hispanic	3 (2.1%)	0 (0.0%)	3 (1.6%)	
Asian	2 (1.4%)	0 (0.0%)	2 (1.1%)	
Medical History				
High Blood Pressure	68 (48.2%)	6 (12.8%)	74 (39.4%)	< 0.001
Hyperlipidemia	52 (36.9%)	3 (6.4%)	55 (29.3%)	< 0.001
Diabetes Mellitus	45 (31.9%)	1 (2.1%)	46 (24.5%)	< 0.001
Prior Trauma/Abd Surgery	82 (58.2%)	4 (8.5%)	86 (45.7%)	< 0.001
Type of Injury				< 0.001
Penetrating	0 (0.0%)	41 (87.2%)	41 (21.8%)	
Blunt	0 (0.0%)	6 (12.8%)	6 (3.2%)	
Post-Surgical	124 (87.9%)	0 (0.0%)	124 (66.0%)	
None	17 (12.1%)	0 (0.0%)	17 (9.0%)	
Detection of Leak				0.005
CTAP	47 (33.3%)	30 (63.8%)	77 (41.0%)	
HIDA	40 (28.4%)	5 (10.6%)	45 (23.9%)	
MRCP	5 (3.5%)	3 (6.4%)	8 (4.3%)	
PCT/JP Biliary Drainage Increase	24 (17.0%)	7 (14.9%)	31 (16.5%)	
Intra-Op Visualization	5 (3.5%)	0 (0.0%)	5 (2.7%)	
US	5 (3.5%)	0 (0.0%)	5 (2.7%)	
Other	15 (10.6%)	2 (4.3%)	17 (9.0%)	
Lab Values				
Stent Placed	134 (95.7%)	47 (100.0%)	181 (96.8%)	0.15
Stent Exchange	21 (15.0%)	5 (10.6%)	26 (13.9%)	0.45
Time to exchange [months]	1.8 (1.2)	2.6 (1.1)	1.9 (1.2)	0.16
Stent Removal	107 (76.4%)	32 (69.6%)	139 (74.7%)	0.35
Time to stent removal [months]	3.7 (2.7)	3.6 (1.6)	3.7 (2.5)	0.74
Length of time of JP/PTC Drain placement (days)	2.3 (3.8)	3.0 (5.3)	2.6 (4.4)	0.47
IR Involvement	47 (33.3%)	28 (59.6%)	75 (39.9%)	0.001
IR Procedure	28 (58%)	24 (86%)	52 (68%)	0.013
IR Drain Exchange?	31 (67%)	19 (68%)	50 (68%)	0.97
Surgical Involvement	7 (5.0%)	3 (6.4%)	10 (5.3%)	0.94
LOS [days]	12.4 (17.0)	21.3 (15.9)	14.6 (17.1)	0.002
Grade				< 0.001
Low	16 (11.3%)	1 (2.1%)	17 (9.0%)	
High	20 (14.2%)	20 (42.6%)	40 (21.3%)	
Unspecified	105 (74.5%)	26 (55.3%)	131 (69.7%)	
Strasberg Classification				
A	62 (65.0%)			
D	25 (26.0%)			
E1	8 (8.0%)			
E2	1 (1.0%)			
Death	7 (5.0%)	0 (0.0%)	7 (3.7%)	0.12
30 day mortality	0 (0.0%)	0 (0.0%)	0 (0.0%)	
60 day mortality	4 (57.1%)	0 (0.0%)	0 (0.0%)	
Related to ERCP or hospitalization	3 (42.9%)	0 (0.0%)	0 (0.0%)	
Complications	5 (3.5%)	2 (4.3%)	7 (3.7%)	0.82
Type of complication				0.22
Abscess	1 (20%)	0 (0%)	1 (14%)	
Biliary strictures	2 (40%)	0 (0%)	2 (29%)	
Bleeding	0 (0%)	1 (50%)	1 (14%)	
Hepatic infarction	0 (0%)	1 (50%)	1 (14%)	
Pancreatitis	1 (20%)	0 (0%)	1 (14%)	
Biloma	1 (20%)	0 (0%)	1 (14%)	

Data are presented as mean (SD) for continuous measures, and n (%) for categorical measures

^*^Traumatic includes Blunt and penetrating and non-traumatic include surgical and medical causes

### Data Collection

Data were extracted through a review of the medical charts. The demographic information obtained included age, sex, and comorbidities. The reason for ERCP was categorized into traumatic and non-traumatic etiologies. Traumatic etiologies that were included were gunshot wounds and motor vehicle accidents. Non-traumatic etiologies were categorized as follows: post-cholecystectomy, post-liver transplant, post-hepatectomy, after other surgical procedure, in relation to a medical condition (e.g. cholecystitis, cholangitis, choledocholithiasis, pancreatitis). Patients that were found to have a bile leak on ERCP were included. The grade of the bile leak was classified as follows: high grade, low grade, unspecified. This definition of injury grade was based on the classification system defined by Sandha et al. in 2004. Low-grade injuries were defined as a leak that was visible in cholangiography from the distal part of the common bile duct only after complete opacification of the intrahepatic biliary radicals. High-grade injuries were defined as leaks seen before complete intrahepatic opacification [12]. Laboratory studies for each patient were collected, including white blood cell count, hemoglobin, serum creatinine, aspartate aminotransferase, alanine aminotransferase, and alkaline phosphatase. Whether patients underwent stent placement or stent exchange was also recorded, including the time to stent exchange if applicable. Hospital length of stay (LOS) was assessed. Stent removal and time for stent removal were also acquired. Non-endoscopic therapy involvement was examined. If appropriate, we specified as to if a drain via percutaneous transhepatic cholangiogram (PTC) or Jackson-Pratt (JP) drain was placed.

### Outcomes

Our primary outcomes were technical success and clinical success. We defined technical success as successful stent placement on initial ERCP. We defined clinical success as overall resolution of leak as established by resolution on imaging or ERCP that eventually led to removal of the bile duct stent. Our secondary outcomes were stent exchange due to persistent bile leak on repeat ERCP, non-endoscopic interventions, hospital LOS, death, and complications. This study was conducted and reported in accordance with the STROBE (Strengthening the Reporting of Observational Studies in Epidemiology) guidelines. This study was approved by the University of Alabama Institutional Review Board.

### Statistical Analysis

Data are presented using the descriptive statistics of the mean ± standard deviation for linear variables and frequency percentage for categorical variables. Categorical variables were compared between the groups using Chi-square and Fisher’s exact test. The analyses were two sided and performed using at a significance level of 5%. For this analysis, we compared demographic and clinical characteristics between patients with traumatic injury and those with non-traumatic etiologies, to identify factors associated with traumatic mechanism. All analyses were performed using SAS, version 9.4 (SAS Institute Inc., Cary, NC, USA).

## Results

**Fig. 1:**
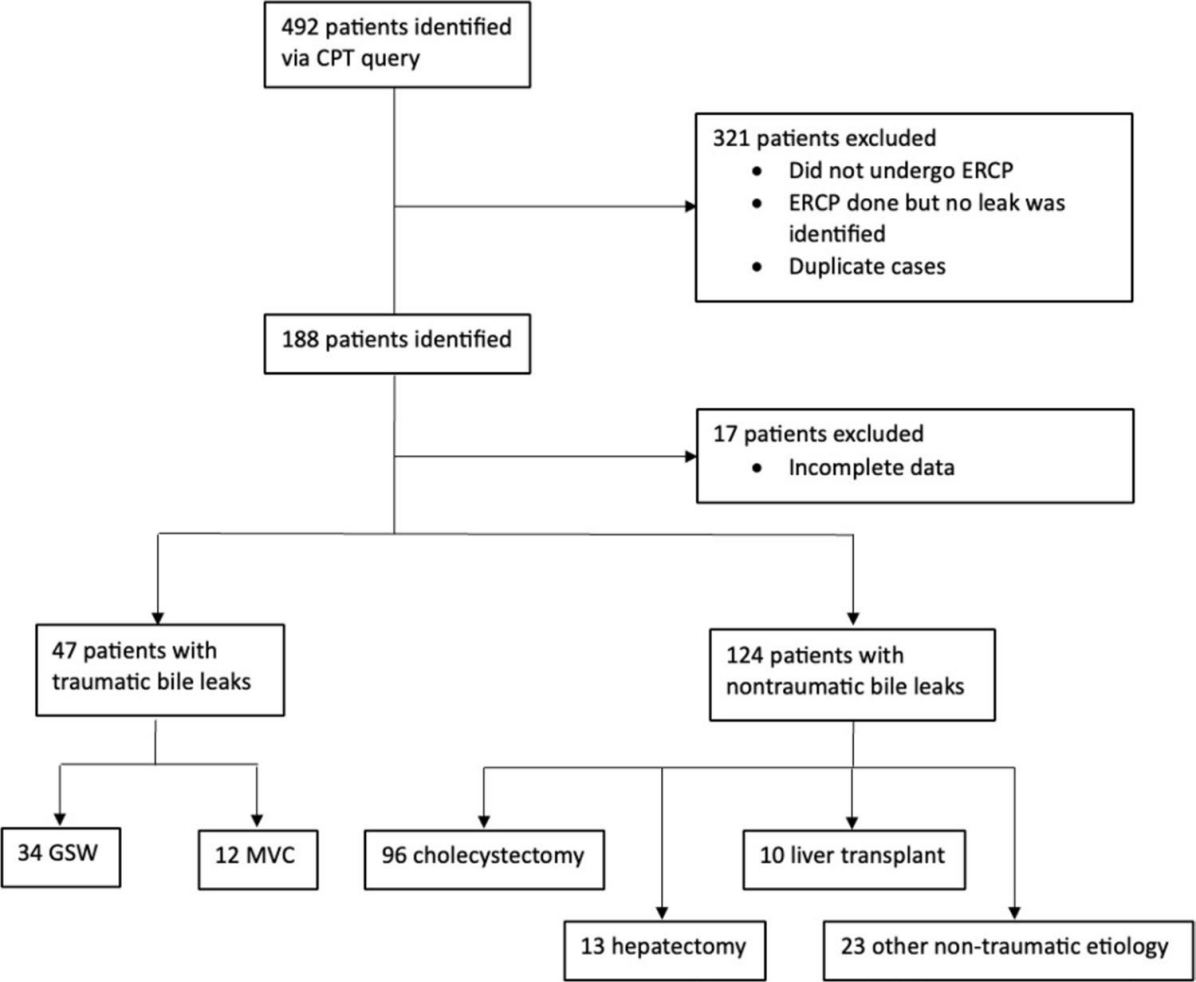
Flow Diagram of Patient Selection for Traumatic and Non-Traumatic Bile Leaks. Caption**:** This flow diagram illustrates the patient selection process for the study, starting from 492 patients identified via CPT query

The study included 188 patients with bile duct leaks, identified from an initial query of 492 cases based on CPT codes. Of these, 141 patients were classified as non-traumatic, and 47 were traumatic cases (Fig. 1​). Data depuration steps excluded patients without confirmed bile leaks, duplicates, and those who did not undergo ERCP.

### Demographics and Medical History

Non-traumatic injuries were more prevalent (75%, n = 141), and patients in this group were significantly older than their traumatic counterparts (mean age 54.4 ± 17.7 vs 30.0 ± 13.4 years; p < 0.001; Table 1​). Age categories showed that 87.2% of traumatic cases were under 40 years, compared to only 27.7% in non-traumatic injuries. Traumatic injuries predominantly affected males (83.0%, n = 39) compared to non-traumatic cases, where females accounted for the majority (53.2%, n = 75; p < 0.001). Racial distribution revealed a higher proportion of Black patients in the traumatic group (68.1%, n = 32) compared to non-traumatic cases, which were predominantly White (76.6%, n = 108; p < 0.001). Non-traumatic injuries were associated with higher prevalence of comorbid conditions, including hypertension (48.2% vs 12.8%; p < 0.001), hyperlipidemia (36.9% vs 6.4%; p < 0.001), and diabetes mellitus (31.9% vs 2.1%; p < 0.001). Prior trauma or abdominal surgery was reported in 58.2% of non-traumatic cases compared to 8.5% in the traumatic group (p < 0.001; Table 1​).

### Type of Injury and Detection of Bile Leaks

**Fig. 2:**
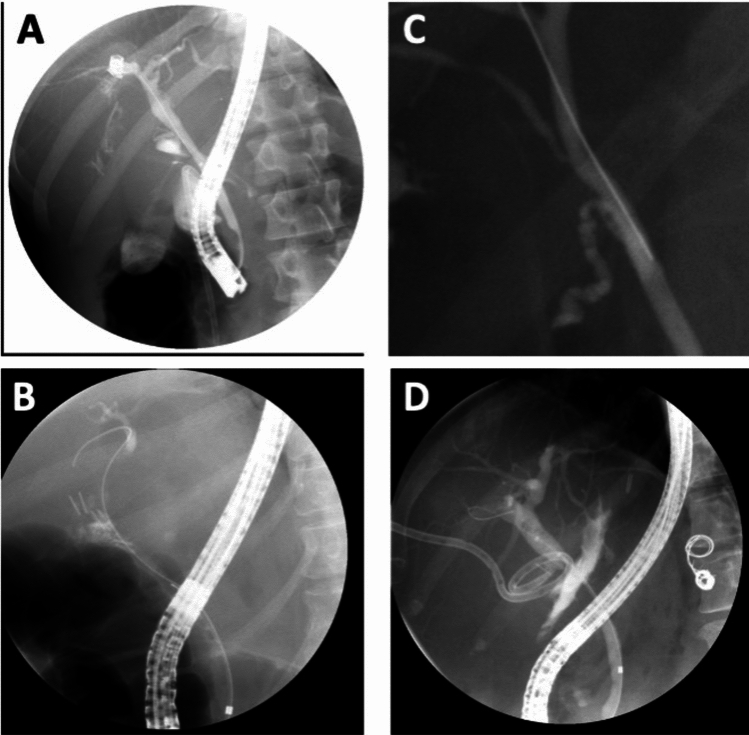
Representative Cholangiogram Images of Bile Duct Leaks from Various Etiologies. Caption: Fig. 1. **A** Bile duct leak secondary to a gun-shot wound as seen on cholangiogram. **B** Bile duct leak after a cholecystectomy as seen on cholangiogram. **C** Bile duct leak secondary to a motor vehicle collision as seen on cholangiogram. **D** Bile duct leak secondary to a hepatectomy as seen on cholangiogram

Traumatic injuries were categorized as penetrating (87.2%, n = 41) or blunt (12.8%, n = 6), with no surgical causes in this group. In contrast, 87.9% (n = 124) of non-traumatic cases were post-surgical, with the remaining classified as medical etiologies or unknown (Table 1​). Bile leaks were detected using a variety of modalities, with CTAP being the most common overall (41%, n = 77). CTAP was used more frequently in traumatic cases (63.8%, n = 30) than non-traumatic ones (33.3%, n = 47; p = 0.005). HIDA scans were more common in non-traumatic cases (28.4% vs 10.6%; p = 0.005), and MRCP, PCT/JP biliary drainage increases, and intraoperative visualizations were infrequently used across both groups (Table 1). Representative cholangiograms illustrating bile duct leaks from various etiologies are shown in Fig. 2.

### Reason for ERCP

The primary reasons for ERCP in non-traumatic cases were post-cholecystectomy bile leaks (68.1%, n = 96) and post-liver transplant leaks (7.1%, n = 10), while traumatic cases were predominantly due to gunshot wounds (72.3%, n = 34) and motor vehicle collisions (25.5%, n = 12; Supplement Table 2​)﻿. Other less frequent indications included leaks related to pancreatitis or medical conditions.

**Table 2:** Univariate and Multivariate logistic Regression Models for the odds of having traumatic Injury vs Non-traumatic Injuries

	Univariate Logistic Regression				Multivariate Logistic Regression			
	OR	[95% CI]		p-value	aOR	[95% CI]		p-value
Age < 40	14.22	6.08	33.26	< 0.001	19.75	6.25	62.39	< 0.001
Gender: Male	5.54	2.42	12.70	< 0.001	6.45	2.11	19.67	0.001
Race: Black	8.61	4.11	18.04	< 0.001	3.41	1.22	9.55	0.019
Stent exchange	0.67	0.24	1.90	0.457	0.41	0.07	2.35	0.32
Stent removal	0.70	0.34	1.48	0.354	0.47	0.15	1.41	0.178
IR Involvement	2.95	1.49	5.82	0.002	2.05	0.73	5.75	0.171
Grade: High vs Low	16.00	1.93	132.40	0.01	15.75	1.03	240.49	0.047
Complications	1.21	0.23	6.45	0.824	0.45	0.02	8.73	0.599

Univariate and multivariate logistic regression models examining the odds of having a traumatic injury versus a non-traumatic injury. Odds ratios (OR), adjusted odds ratios (aOR), 95% confidence intervals (CI), and p-values are presented for each variable. Significant associations are observed with age under 40, male gender, Black race, and high-grade injuries

### Procedure Characteristics

A stent was placed during ERCP in 96.8% of cases (technical success), with no significant difference between traumatic (100%, n = 47) and non-traumatic (95.7%, n = 134) groups (p = 0.15; Table 1). Stent exchange was required in 13.9% (n = 26) of patients, with a mean time to exchange of 2.6 ± 1.1 months in traumatic cases versus 1.8 ± 1.2 months in non-traumatic ones (p = 0.16). All patients who underwent biliary stent placement had a biliary sphincterotomy performed at the time of first stent placement, consistent with our institutional standard of practice. All stents were plastic, with diameters ranging from 7 to 10 Fr and lengths from 9 to 12 cm. The majority of patients received a single stent; however, 19 patients (13.5%) in the non-traumatic group and 11 patients (23.4%) in the traumatic group required placement of two stents. Bridging of the leak site was achieved in [n = 119, 96%] of non-traumatic cases and [n = 47, 100%] of traumatic cases (p = 0.374).

Clinical success with overall resolution that lead to stent removal occurred in 74.7% of cases (n = 139), with a similar proportion between groups (traumatic: 69.6%, n = 32; non-traumatic: 76.4%, n = 107; p = 0.35). The average time to removal was 3.7 ± 2.5 months, with no significant difference between groups (3.7 ± 2.7 vs 3.6 ± 1.6, p = 0.74; in the traumatic vs non-traumatic groups respectively, Table 1​). Interventional radiology (IR) involvement was significantly higher in traumatic cases (59.6%, n = 28) compared to non-traumatic cases (33.3%, n = 47; p = 0.001). Among those requiring IR, procedures such as drainage or drain exchange were more commonly performed in traumatic cases (86% vs 58%; p = 0.013). Hospital length of stay (LOS) was markedly longer in traumatic cases (21.3 ± 15.9 days vs 12.4 ± 17.0 days; p = 0.002; Table 1​).

### Laboratory Values

Laboratory findings highlighted more severe abnormalities in traumatic cases. White blood cell counts were higher in traumatic injuries (16.1 ± 9.4 × 10^9/L) compared to non-traumatic cases (11.2 ± 7.4 × 10^9/L; p < 0.001). Hemoglobin levels were lower in traumatic cases (9.5 ± 2.1 g/dL vs 10.8 ± 2.0 g/dL; p < 0.001). Liver enzyme levels, including AST (95.6 ± 139.7 U/L vs 51.8 ± 71.9 U/L; p = 0.006) and ALT (125.9 ± 163.7 U/L vs 64.9 ± 107.1 U/L; p = 0.004), were also elevated in traumatic cases (Supplement Table 3).

### Severity and Complications

High-grade bile leaks were significantly more common in traumatic cases (42.6%, n = 20) than in non-traumatic ones (14.2%, n = 20; p < 0.001). Low-grade leaks were predominantly observed in non-traumatic cases (11.3% vs 2.1%; p < 0.001; Table 1). According to the Strasberg classification, cholecystectomy patients injuries exhibited high proportions of grades A and D injuries (65% and 28%, respectively) compared to non-traumatic injuries (32.6% and 7.8%, respectively; p = 0.014). In the traumatic cohort, associated injuries were common. Documented injuries included liver lacerations [n = 23, 48.9%], vascular injury [n = 4, 8.5%], hollow viscus injury [n = 3, 6.4%], and other organ involvement including diaphragm [n = 2, 4.3%], pancreas [n = 1, 2.1%], spleen [n = 4, 8.5%], lung [n = 15, 31.9%], and cardiac injury [n = 0, 0.0%]. Other organ lacerations were also recorded [n = 5]. These findings highlight that bile duct injuries in trauma patients rarely occur in isolation and are frequently accompanied by significant multiorgan involvement. A complete breakdown of associated injuries is provided in Supplement Table 6.

Surgical involvement was similar between groups (11% in both n = 7 in the non-traumatic group and n = 3 in the traumatic group; p = 0.94). In the non-traumatic group, interventions included surgical drain placement (n = 1), open bile duct reconstruction (n = 3), and hepaticojejunostomy to the right bile duct (n = 3); in the traumatic injury group, interventions included exploratory laparotomy with hepatorrhaphy (n = 1), perihepatic surgical drain placement (n = 1), and surgical drain replacement (n = 1).

Overall complication rates were low, with 3.5% (n = 5) in non-traumatic and 4.3% (n = 2) in traumatic cases (p = 0.82). Complications included biliary strictures, bleeding, abscess formation, and pancreatitis. Deaths were observed exclusively in the non-traumatic group (5.0%, n = 7; p = 0.12; Table 1​). Of these deaths, none occurred within 30 days, 4 (57%) within 60 days, and 3 (43%) after 90 days of the procedure. Additionally, 4 (57.1%) occurred during the same hospitalization as the procedure, while 3 (42.9%) were determined to be unrelated to the procedure or the current cause of hospitalization.

### Logistic Regression Model

Multivariate logistic regression identified significant predictors of traumatic bile injuries. Younger age (< 40 years) increased the odds of traumatic injury nearly 20-fold (aOR 19.75, 95% CI 6.25–62.39; p < 0.001), while male gender (aOR 6.45, 95% CI 2.11–19.67; p = 0.001) and Black race (aOR 3.41, 95% CI 1.22–9.55; p = 0.019) were also independent predictors. High-grade leaks were strongly associated with traumatic injuries (aOR 15.75, 95% CI 1.03–240.49; p = 0.047; Table 2).

### Sub-analysis of Surgical Versus Traumatic Etiologies

A focused analysis comparing surgical (n = 124) and traumatic (n = 47) injuries revealed consistent trends. Surgical injuries had a higher prevalence of comorbidities, while traumatic cases were younger and predominantly male. LOS remained significantly longer in traumatic injuries (21.3 ± 15.9 days vs 11.3 ± 13.8 days; p < 0.001). The need for IR intervention was higher in traumatic injuries (59.6% vs 33.9%; p = 0.002), with more frequent use of drainage procedures (Supplement Table 4​). Characteristics and outcomes for each ethology can be found in supplement Table 4 and 5.

## Discussion

To our knowledge, this is one of the first studies providing a detailed and comprehensive analysis of the management of bile leaks, comparing traumatic and non-traumatic etiologies. Our results show that trauma patients were more commonly younger and male. Trauma patients also exhibited some more concerning laboratory values and higher-grade injuries, indicating a higher level of severity in these patients compared to non-traumatic injuries. Hospital stay was significantly longer in traumatic cases, reflecting greater injury severity and complexity, including a higher need for interventional radiology. This finding is expected, as many of these patients likely sustained multiorgan trauma requiring additional surgical interventions and ICU care. Despite these differences between traumatic and nontraumatic bile leaks, however, our results underscore high rates of technical and clinical success in both traumatic and nontraumatic bile leaks, with these outcomes as well as mortality and complications not differing between both groups.

Our study highlights that clinical parameters in traumatic bile leaks tend to be more severe compared to nontraumatic bile leaks. Given the nature of penetrating and blunt traumatic injuries, the differences we see in laboratory studies and grade of injury can be rationalized. Although significant injuries can also occur secondary to operative intervention and in the setting of medical conditions such as acute cholangitis, gunshot wounds and motor vehicle accidents injuries are less predictable and more diffuse in nature, providing comprehensibility to our results. Despite these differences in clinical parameters, technical and clinical success is high regardless of bile leak etiology.

Although technical and clinical success were high in both groups in our study, there were cases in which success was not achieved. Studies have been done to examine and elucidate predictors of success in endoscopic management of bile leaks. In one retrospective analysis of 101 bile leak patients, cystic duct leak and leak-bridging drainage were factors positively associated with ERCP success whereas systemic inflammatory response syndrome (SIRS) and high-grade leaks were found to be negative predictors of ERCP success. Most patients included in the study had bile leak secondary to cholecystectomy with only 4% secondary to trauma which was not further defined [13]. Another retrospective study looking at 177 patients that suffered from bile leaks after cholecystectomy demonstrated that biliary stent placement and cystic duct leaks or higher predictors of leak resolution [14]. A retrospective study looking at 58 patients underwent ERCP for bile leak after hepatobiliary surgery and revealed that bile leak severity (large leak versus small leak) was an independent factor associated with successful endoscopic management [15]. Further research needs to be conducted to delineate predictors of success for endoscopic management of traumatic bile leaks.

Our study establishes a significantly higher need for non-endoscopic intervention traumatic compared to the nontraumatic bile duct injuries. Clinical success was high and did not significantly differ between both groups but some patients required non-endoscopic intervention in addition to ERCP to aid in management of their bile leaks. Studies have established the efficacy of the non-endoscopic interventions in the treatment of bile leaks. A retrospective study analyzing 101 percutaneous treatments for bile leaks treated with PTC drains showed technical success achieved in 93% of patients with no recurrence of leak in 94% of patients [16]. Another retrospective study looking at 74 patients who also underwent PTC drain placement showed 91.8% technical success and 80.8% clinical success rates for management of bile leaks, with 93.2% of leaks in this study being post-surgical [17]. The average rate of major complications for PTC drain placement is estimated to be 2.5% [18]. Furthermore, a retrospective study examining JP drain placement for the management of bile leaks secondary to open and laparoscopic cholecystectomy in 40 patients showed that these drains were an effective adjunctive tool for the diagnosis of biliary injuries [19]. A large majority of prior studies have examined the usage of non-endoscopic therapies for the management of bile leaks that we defined in our study as non-traumatic. Our findings contribute to the literature by displaying efficacy of the ERCP and non-endoscopic in the management of bile leaks.

Recent data regarding the incidence of gunshot wounds and motor vehicle collisions highlights the importance of our study and findings from a public health standpoint. A study that calculated population-based death rates (per 100,000 population) using data from the International Road Traffic and Accident Database showed that the U.S. had the highest population-based road traffic death rate (11.1) compared with 28 other high-income countries in 2019 [20]. Regarding firearm deaths, 48,830 people in the U.S. lost their lives from firearms in 2021, of which 53.8% were suicides and 42.9% were homicides [21]. An observational study of patients hospitalized for GSW in the National Inpatient Sample between 2004 and 2013 found that each year approximately 30,000 patients are hospitalized for GSW, with most (6**3**%) due to assaults [22]. In a 2020 study, the lifetime risk of death from firearms and motor vehicle accidents was calculated to be 0.93% and 0.92%, respectively [23]. Given the high frequency rate of these events that we have seen in recent years, these statistics highlight the importance of understanding differences in therapeutic interventions and outcomes for patients with bile duct injuries secondary to traumatic etiologies.

Our study has a few limitations. First, this was a retrospective study, resulting in limitations and inconsistencies in the data availability and interpretation. Second, although a larger subset of patients were included compared to some prior studies examining traumatic and nontraumatic bile leaks, this was still a small sample size of less than 200 patients. Third, all procedures were performed at a single institution, thus limiting our ability to generalize the results to a larger population subset. Fourth, some patients were lost to follow-up for repeat ERCP or imaging and subsequent stent removal, possibly confounding data regarding clinical success.

Finally, several patients in the traumatic group had other injuries and medical conditions for which they needed to remain hospitalized. Due to this, length of stay in these patients may not accurately reflect differences between nontraumatic bile leaks, as they had other indications for continued hospitalization outside of the bile duct leak itself. Another limitation of our study is that we did not capture Injury Severity Scores (ISS), as our dataset was derived from an ERCP-focused database rather than a trauma registry. This limited our ability to provide a standardized severity comparison across trauma patients, though we did include associated organ injury data to provide clinical context.

However, despite these limitations, our study revealed traumatic bile leaks as an important etiology of complex bile leaks, with a significantly higher number requiring combined treatment modalities in comparison to nontraumatic bile leaks. Nevertheless, technical and clinical success as well as mortality and adverse events did not significantly differ between groups. Although this study does not provide novel insights into the technical role of ERCP, it contributes real-world data on ERCP outcomes specifically in patients with traumatic bile leak, particularly in settings with a high burden of penetrating injuries, despite being limited by its single-center, retrospective design. Our study highlights that tailored management strategies outside of ERCP are needed to address the distinct clinical characteristics that we see in traumatic and non-traumatic bile leaks.

## Supplementary Information

Below is the link to the electronic supplementary material.

Supplementary file1 (DOCX 37 KB)

Supplementary file2 (DOCX 54 KB)

## Data Availability

Data will be available upon request to the corresponding author.
