# Supplementary material for: Unveiling the Complexity in the Management and Outcomes of Traumatic Versus Non-Traumatic Bile Leaks—A Comparative Analysis
Source: Dig Dis Sci. 2025 Nov 22;71(5):2015–23. doi: 10.1007/s10620-025-09550-6 (PMC13201356; doi:10.1007/s10620-025-09550-6)
Supplement: Supplementary file 1 — Supplementary file1 (DOCX 37 KB) [file 10620_2025_9550_MOESM1_ESM.docx]

STROBE Statement—checklist of items that should be included in reports of observational studies

|  | Item No. | Recommendation | Page  No. | Relevant text from manuscript |
| --- | --- | --- | --- | --- |
| **Title and abstract** | 1 | (*a*) Indicate the study’s design with a commonly used term in the title or the abstract | 1 | A comparative analysis |
|  |  | (*b*) Provide in the abstract an informative and balanced summary of what was done and what was found | 2 | **Abstract** |
| Introduction | | | |  |
| Background/rationale | 2 | Explain the scientific background and rationale for the investigation being reported | 3 | recent literature providing a comparative analysis of patients requiring ERCP for the management of bile duct leaks of various traumatic and non-traumatic causes is scarce. |
| Objectives | 3 | State specific objectives, including any prespecified hypotheses | 4 | We aimed to provide a comprehensive examination comparing the differences in management complexity and outcomes of patients with traumatic versus non-traumatic bile leaks. |
| Methods | | | |  |
| Study design | 4 | Present key elements of study design early in the paper | 4 | We performed a retrospective review of patients who underwent ERCP for suspected biliary leak |
| Setting | 5 | Describe the setting, locations, and relevant dates, including periods of recruitment, exposure, follow-up, and data collection | 4 | between May 22, 2017, and October 9, 2023, at the University of Alabama at Birmingham, an academic tertiary care center |
| Participants | 6 | (*a*) *Cohort study*—Give the eligibility criteria, and the sources and methods of selection of participants. Describe methods of follow-up  *Case-control study*—Give the eligibility criteria, and the sources and methods of case ascertainment and control selection. Give the rationale for the choice of cases and controls  *Cross-sectional study*—Give the eligibility criteria, and the sources and methods of selection of participants | 4 | following diagnoses: established bile leak, suspected bile leak, follow-up bile leak, biliary leak on CT, and pancreatic duct leak. These corresponded to the following Current Procedural Terminology (CPT) codes: 51.36, 51.37, 51.39, 51.71, 51.72, 51.84, 52.98, and 51.79 |
|  |  | (*b*) *Cohort study*—For matched studies, give matching criteria and number of exposed and unexposed  *Case-control study*—For matched studies, give matching criteria and the number of controls per case |  |  |
| Variables | 7 | Clearly define all outcomes, exposures, predictors, potential confounders, and effect modifiers. Give diagnostic criteria, if applicable | 5 | The primary outcomes were technical and clinical success. Technical success was defined as successful stent placement during the initial ERCP. We defined clinical success as the overall resolution of the leak, as established by the resolution on imaging or ERCP. Our secondary outcomes were stent exchange due to persistent bile leak on repeat ERCP, non-endoscopic intervention, hospital LOS, death, and complications |
| Data sources/ measurement | 8* | For each variable of interest, give sources of data and details of methods of assessment (measurement). Describe comparability of assessment methods if there is more than one group | *4* | *The study population was identified by querying the institution’s Provation database using the following diagnoses* |
| Bias | 9 | Describe any efforts to address potential sources of bias | 6 | To reduce potential sources of bias, we applied strict inclusion and exclusion criteria, conducted multi-stage data cleaning, and used standardized definitions for clinical variables. |
| Study size | 10 | Explain how the study size was arrived at |  | Entire population in the time period. |

Continued on next page

| Quantitative variables | 11 | Explain how quantitative variables were handled in the analyses. If applicable, describe which groupings were chosen and why | 6 | Statistical Analysis  Data are presented using the descriptive statistics of the mean +/- standard deviation for linear variables and frequency percentage for categorical variables |
| --- | --- | --- | --- | --- |
| Statistical methods | 12 | (*a*) Describe all statistical methods, including those used to control for confounding | 6 | Categorical variables were compared between the groups using Chi-square and Fisher’s exact tests. The analyses were two-sided and were performed at a significance level of 5%. All analyses were performed using SAS, version 9.4 (SAS Institute, Inc., Cary, NC, USA). |
|  |  | (*b*) Describe any methods used to examine subgroups and interactions | 6 | Categorical variables were compared between the groups using Chi-square and Fisher’s exact tests. |
|  |  | (*c*) Explain how missing data were addressed | 6 | Missing data were assessed for each variable, and cases with incomplete key outcome or exposure data were excluded from the final analysis. |
|  |  | (*d*) *Cohort study*—If applicable, explain how loss to follow-up was addressed  *Case-control study*—If applicable, explain how matching of cases and controls was addressed  *Cross-sectional study*—If applicable, describe analytical methods taking account of sampling strategy |  |  |
|  |  | (*e*) Describe any sensitivity analyses |  |  |
| Results | | | | |
| Participants | 13* | (a) Report numbers of individuals at each stage of study—eg numbers potentially eligible, examined for eligibility, confirmed eligible, included in the study, completing follow-up, and analysed | 6 | This study included 188 patients with bile duct leaks identified from an initial query of 492 cases based on CPT codes. Of these, 141 were classified as non-traumatic and 47 as traumatic (Figure 1). The data depuration steps excluded patients without confirmed bile leaks, duplicates, and those who did not undergo ERCP. |
|  |  | (b) Give reasons for non-participation at each stage | Figure 1 |  |
|  |  | (c) Consider use of a flow diagram | Figure 1 |  |
| Descriptive data | 14* | (a) Give characteristics of study participants (eg demographic, clinical, social) and information on exposures and potential confounders | 7 | The age categories showed that 87.2% of traumatic cases were under 40 years of age, compared to only 27.7% of non-traumatic injuries. Traumatic injuries predominantly affected males (83.0%, n = 39) compared to non-traumatic injuries, where females accounted for the majority (53.2%, n = 75; p < 0.001). Racial distribution revealed a higher proportion of black patients in the traumatic group (68.1%, n = 32) compared to non-traumatic cases, which were predominantly white (76.6%, n = 108; p < 0.001) |
|  |  | (b) Indicate number of participants with missing data for each variable of interest | Table 1 |  |
|  |  | (c) *Cohort study*—Summarise follow-up time (eg, average and total amount) |  |  |
| Outcome data | 15* | *Cohort study*—Report numbers of outcome events or summary measures over time |  |  |
|  |  | *Case-control study—*Report numbers in each exposure category, or summary measures of exposure |  |  |
|  |  | *Cross-sectional study—*Report numbers of outcome events or summary measures | *9* | *The overall complication rates were low, with 3.5% (n = 5) in non-traumatic cases and 4.3% (n = 2) in traumatic cases (p = 0.82).* |
| Main results | 16 | (*a*) Give unadjusted estimates and, if applicable, confounder-adjusted estimates and their precision (eg, 95% confidence interval). Make clear which confounders were adjusted for and why they were included | 9 | Multivariate logistic regression was used to identify significant predictors of traumatic bile injury. Younger age (<40 years) increased the odds of traumatic injury nearly 20-fold (aOR 19.75, 95% CI 6.25–62.39; p < 0.001), while male sex (aOR 6.45, 95% CI 2.11–19.67; p = 0.001) and black race (aOR 3.41, 95% CI 1.22–9.55; p = 0.019) were also independent predictors. High-grade leaks were strongly associated with traumatic injuries (aOR 15.75, 95% CI 1.03–240.49; p = 0.047; Table 2). |
|  |  | (*b*) Report category boundaries when continuous variables were categorized | NA |  |
|  |  | (*c*) If relevant, consider translating estimates of relative risk into absolute risk for a meaningful time period | NA |  |

Continued on next page

| Other analyses | 17 | Report other analyses done—eg analyses of subgroups and interactions, and sensitivity analyses |  |  |
| --- | --- | --- | --- | --- |
| Discussion | | | | |
| Key results | 18 | Summarise key results with reference to study objectives |  |  |
| Limitations | 19 | Discuss limitations of the study, taking into account sources of potential bias or imprecision. Discuss both direction and magnitude of any potential bias |  |  |
| Interpretation | 20 | Give a cautious overall interpretation of results considering objectives, limitations, multiplicity of analyses, results from similar studies, and other relevant evidence |  |  |
| Generalisability | 21 | Discuss the generalisability (external validity) of the study results |  |  |
| Other information | |  | | |
| Funding | 22 | Give the source of funding and the role of the funders for the present study and, if applicable, for the original study on which the present article is based |  |  |

*Give information separately for cases and controls in case-control studies and, if applicable, for exposed and unexposed groups in cohort and cross-sectional studies.

**Note:** An Explanation and Elaboration article discusses each checklist item and gives methodological background and published examples of transparent reporting. The STROBE checklist is best used in conjunction with this article (freely available on the Web sites of PLoS Medicine at http://www.plosmedicine.org/, Annals of Internal Medicine at http://www.annals.org/, and Epidemiology at http://www.epidem.com/). Information on the STROBE Initiative is available at www.strobe-statement.org.
