# Supplementary material for: Unveiling the Complexity in the Management and Outcomes of Traumatic Versus Non-Traumatic Bile Leaks—A Comparative Analysis
Source: Dig Dis Sci. 2025 Nov 22;71(5):2015–23. doi: 10.1007/s10620-025-09550-6 (PMC13201356; doi:10.1007/s10620-025-09550-6)
Supplement: Supplementary file 2 — Supplementary file2 (DOCX 54 KB) [file 10620_2025_9550_MOESM2_ESM.docx]

**Supplement 1. CTP procedure codes**

| **Code** | **Procedure** |
| --- | --- |
| 51.36 | Choledochoenterostomy |
| 51.37 | Anastomosis of hepatic duct to gastrointestinal tract |
| 51.39 | Other bile duct anastomosis |
| 51.71 | Simple suture of common bile duct |
| 51.72 | Choledochoplasty |
| 51.84, 52.98 | ERCP |
| 51.79 | Repair of other bile ducts |

Data are presented as mean (SD) for continuous measures, and n (%) for categorical measures.

*Traumatic includes: Blunt and penetrating and non-traumatic include surgical and medical causes.

**Supplement Table 2. Reason for ERCP in non-traumatic vs Traumatic Patients.**

|  | **Non-traumatic** | **Traumatic Injury** | **Total** | **p-value** |
| --- | --- | --- | --- | --- |
| **Reason for ERCP** |  |  |  | **<0.001** |
| GSW | 0 ( 0.0%) | 34 (72.3%) | 34 (18.1%) |  |
| MVC | 0 ( 0.0%) | 12 (25.5%) | 12 ( 6.4%) |  |
| Cholecystectomy | 96 (68.1%) | 0 ( 0.0%) | 96 (51.1%) |  |
| Liver Transplant | 10 ( 7.1%) | 0 ( 0.0%) | 10 ( 5.3%) |  |
| Hepatectomy | 13 ( 9.2%) | 0 ( 0.0%) | 13 ( 6.9%) |  |
| Other Surgery | 5 ( 3.5%) | 0 ( 0.0%) | 5 ( 2.7%) |  |
| Pancreatitis | 7 ( 5.0%) | 0 ( 0.0%) | 7 ( 3.7%) |  |
| Other Medical Condition | 10 ( 7.1%) | 1 ( 2.1%) | 11 ( 5.9%) |  |

Data are presented as mean (SD) for continuous measures, and n (%) for categorical measures.

*Traumatic includes: Blunt and penetrating and non-traumatic include surgical and medical causes.

**Supplement 3. Laboratory values in non-traumatic vs traumatic injuries.**

|  | **Non-traumatic** | **Traumatic Injury** | **Total** | **p-value** |
| --- | --- | --- | --- | --- |
| White Blood Cells [x 10^9 Cells/L] | 11.2 (7.4) | 16.1 (9.4) | 12.4 (8.2) | <0.001 |
| Hemoglobin [g/dL] | 10.8 (2.0) | 9.5 (2.1) | 10.5 (2.1) | <0.001 |
| Creatinine (mg/dL) | 1.0 (0.7) | 1.0 (0.9) | 1.0 (0.7) | 0.76 |
| AST [Units/L] | 51.8 (71.9) | 95.6 (139.7) | 62.8 (95.1) | 0.006 |
| ALT [Units/L] | 64.9 (107.1) | 125.9 (163.7) | 80.3 (126.1) | 0.004 |
| ALP [U/L] | 163.0 (146.6) | 179.5 (146.0) | 167.2 (146.2) | 0.51 |

Data are presented as mean (SD) for continuous measures, and n (%) for categorical measures.

*Traumatic includes: Blunt and penetrating and non-traumatic include surgical and medical causes.

**Supplement 4. Sub-analysis only including iatrogenic (surgical) vs traumatic injuries (Blunt/penetrating).**

|  | **Surgical** | **Traumatic** | **p-value** |
| --- | --- | --- | --- |
|  | **N=124** | **N=47** |  |
| Age [years] | 55.3 (17.8) | 30.0 (13.4) | <0.001 |
| Age Category |  |  | <0.001 |
| <18 | 0 ( 0.0%) | 5 (10.6%) |  |
| 18-25 | 8 ( 6.5%) | 15 (31.9%) |  |
| 26-40 | 23 (18.5%) | 19 (40.4%) |  |
| 40-60 | 41 (33.1%) | 6 (12.8%) |  |
| >61 | 52 (41.9%) | 2 ( 4.3%) |  |
| Gender |  |  | <0.001 |
| Female | 64 (51.6%) | 8 (17.0%) |  |
| Male | 60 (48.4%) | 39 (83.0%) |  |
| Race |  |  | <0.001 |
| White | 95 (76.6%) | 15 (31.9%) |  |
| Black | 25 (20.2%) | 32 (68.1%) |  |
| Hispanic | 3 ( 2.4%) | 0 ( 0.0%) |  |
| Asian | 1 ( 0.8%) | 0 ( 0.0%) |  |
| Medical History |  |  |  |
| High Blood Pressure | 59 (47.6%) | 6 (12.8%) | <0.001 |
| Hyperlipidemia | 43 (34.7%) | 3 ( 6.4%) | <0.001 |
| Diabetes Mellitus | 42 (33.9%) | 1 ( 2.1%) | <0.001 |
| Prior Trauma/Abd Surgery | 70 (56.5%) | 4 ( 8.5%) | <0.001 |
| Type of Injury |  |  | <0.001 |
| Penetrating | 0 ( 0.0%) | 41 (87.2%) |  |
| Blunt | 0 ( 0.0%) | 6 (12.8%) |  |
| Post-Surgical | 124 (100.0%) | 0 ( 0.0%) |  |
| Detection of Leak |  |  | 0.009 |
| CTAP | 42 (33.9%) | 30 (63.8%) |  |
| HIDA | 36 (29.0%) | 5 (10.6%) |  |
| MRCP | 4 ( 3.2%) | 3 ( 6.4%) |  |
| PCT/JP Biliary Drainage Increase | 24 (19.4%) | 7 (14.9%) |  |
| Intra-Op Visualization | 5 ( 4.0%) | 0 ( 0.0%) |  |
| US | 3 ( 2.4%) | 0 ( 0.0%) |  |
| Other | 10 ( 8.1%) | 2 ( 4.3%) |  |
| Labs |  |  |  |
| White Blood Cells [x 10^9 Cells/L] | 11.5 (7.7) | 16.1 (9.4) | 0.001 |
| Hemoglobin [g/dL] | 10.8 (2.0) | 9.5 (2.1) | <0.001 |
| Creatinine (mg/dL) | 1.0 (0.7) | 1.0 (0.9) | 0.65 |
| AST [Units/L] | 49.2 (66.2) | 95.6 (139.7) | 0.004 |
| ALT [Units/L] | 67.6 (110.6) | 125.9 (163.7) | 0.008 |
| ALP [U/L] | 165.7 (153.1) | 179.5 (146.0) | 0.6 |
| Stent Placed |  |  | 0.12 |
| No | 6 ( 4.9%) | 0 ( 0.0%) |  |
| Yes | 117 (95.1%) | 47 (100.0%) |  |
| Stent Exchange |  |  | 0.5 |
| No | 105 (85.4%) | 42 (89.4%) |  |
| Yes | 18 (14.6%) | 5 (10.6%) |  |
| Time to exchange [months] | 1.7 (1.1) | 2.6 (1.1) | 0.14 |
| Stent Removal |  |  | 0.25 |
| No | 27 (22.0%) | 14 (30.4%) |  |
| Yes | 96 (78.0%) | 32 (69.6%) |  |
| Time to stent removal [months] | 3.8 (2.7) | 3.6 (1.6) | 0.67 |
| Length of time of JP/PTC Drain placement (days) | 2.5 (4.0) | 3.0 (5.3) | 0.67 |
| IR Involvement |  |  | 0.002 |
| No | 82 (66.1%) | 19 (40.4%) |  |
| Yes | 42 (33.9%) | 28 (59.6%) |  |
| IR Procedure |  |  | 0.023 |
| No | 17 (40%) | 4 (14%) |  |
| Yes | 26 (60%) | 24 (86%) |  |
| IR Drain Exchange? |  |  | 0.65 |
| No | 15 (38%) | 9 (32%) |  |
| Yes | 25 (62%) | 19 (68%) |  |
| LOS [days] | 11.3 (13.8) | 21.3 (15.9) | <0.001 |
| Grade |  |  | <0.001 |
| 0 | 15 (12.1%) | 1 ( 2.1%) |  |
| 1 | 19 (15.3%) | 20 (42.6%) |  |
| 2 | 90 (72.6%) | 26 (55.3%) |  |
| Strasberg Classification |  |  | 0.017 |
| A | 62 (64.6%) |  |  |
| D | 25 (24.0%) |  |  |
| E1 | 8 ( 8.3%) |  |  |
| E2 | 1 ( 1.0%) |  |  |
| Death |  |  | 0.12 |
| No | 118 (95.2%) | 47 (100.0%) |  |
| Yes | 6 ( 4.8%) | 0 ( 0.0%) |  |
| Complications |  |  | 0.95 |
| No | 119 (96.0%) | 45 (95.7%) |  |
| Yes | 5 ( 4.0%) | 2 ( 4.3%) |  |
| Type of complication |  |  | 0.22 |
| Abscess | 1 (20%) | 0 ( 0%) |  |
| Biliary strictures | 2 (40%) | 0 ( 0%) |  |
| Bleeding | 0 ( 0%) | 1 (50%) |  |
| Hepatic infarction | 0 ( 0%) | 1 (50%) |  |
| Pancreatitis | 1 (20%) | 0 ( 0%) |  |
| Biloma | 1 (20%) | 0 ( 0%) |  |

Data are presented as mean (SD) for continuous measures, and n (%) for categorical measures.

*Traumatic includes: Blunt and penetrating and surgical include surgical or iatrogenic .

**Supplement 5. Sub-analysis by ethiology.**

|  | GSW | MVC | Cholecystectomy | Liver Transplant | Hepatectomy | Other Surgery | Pancreatitis | Other Medical Condition | p-value |
| --- | --- | --- | --- | --- | --- | --- | --- | --- | --- |
|  |  |  |  |  |  |  |  |  |  |
|  | N=34 | N=12 | N=96 | N=10 | N=13 | N=5 | N=7 | N=11 |  |
|  |  |  |  |  |  |  |  |  |  |
| Age [years] | 28.9 (11.6) | 34.5 (17.1) | 54.0 (18.7) | 52.0 (12.4) | 65.6 (13.8) | 60.0 (10.3) | 44.1 (17.9) | 47.2 (18.2) | <0.001 |
|  |  |  |  |  |  |  |  |  |  |
| Age group |  |  |  |  |  |  |  |  | <0.001 |
| <18 | 3 ( 9%) | 1 ( 8%) | 0 ( 0%) | 0 ( 0%) | 0 ( 0%) | 0 ( 0%) | 0 ( 0%) | 1 ( 9%) |  |
| 18-25 | 11 (32%) | 4 (33%) | 6 ( 6%) | 1 (10%) | 1 ( 8%) | 0 ( 0%) | 0 ( 0%) | 1 ( 9%) |  |
| 26-40 | 16 (47%) | 3 (25%) | 23 (24%) | 0 ( 0%) | 0 ( 0%) | 0 ( 0%) | 4 (57%) | 3 (27%) |  |
| 40-60 | 3 ( 9%) | 3 (25%) | 30 (31%) | 8 (80%) | 0 ( 0%) | 3 (60%) | 1 (14%) | 2 (18%) |  |
| >61 | 1 ( 3%) | 1 ( 8%) | 37 (39%) | 1 (10%) | 12 (92%) | 2 (40%) | 2 (29%) | 4 (36%) |  |
|  |  |  |  |  |  |  |  |  |  |
| Gender |  |  |  |  |  |  |  |  | <0.001 |
| Female | 3 ( 9%) | 4 (33%) | 51 (53%) | 3 (30%) | 7 (54%) | 3 (60%) | 5 (71%) | 7 (64%) |  |
| Male | 31 (91%) | 8 (67%) | 45 (47%) | 7 (70%) | 6 (46%) | 2 (40%) | 2 (29%) | 4 (36%) |  |
|  |  |  |  |  |  |  |  |  |  |
| Race |  |  |  |  |  |  |  |  | <0.001 |
| White | 6 (18%) | 8 (67%) | 68 (71%) | 10 (100%) | 12 (92%) | 5 (100%) | 5 (71%) | 9 (82%) |  |
| Black | 28 (82%) | 4 (33%) | 24 (25%) | 0 ( 0%) | 1 ( 8%) | 0 ( 0%) | 2 (29%) | 1 ( 9%) |  |
| Hispanic | 0 ( 0%) | 0 ( 0%) | 3 ( 3%) | 0 ( 0%) | 0 ( 0%) | 0 ( 0%) | 0 ( 0%) | 0 ( 0%) |  |
| Asian | 0 ( 0%) | 0 ( 0%) | 1 ( 1%) | 0 ( 0%) | 0 ( 0%) | 0 ( 0%) | 0 ( 0%) | 1 ( 9%) |  |
|  |  |  |  |  |  |  |  |  |  |
| High Blood Pressure | 2 ( 6%) | 4 (33%) | 42 (44%) | 5 (50%) | 7 (54%) | 5 (100%) | 3 (43%) | 6 (55%) | <0.001 |
|  |  |  |  |  |  |  |  |  |  |
| Hyperlipidemia | 1 ( 3%) | 2 (17%) | 33 (34%) | 3 (30%) | 7 (54%) | 0 ( 0%) | 3 (43%) | 6 (55%) | 0.001 |
|  |  |  |  |  |  |  |  |  |  |
| Diabetes Mellitus | 0 ( 0%) | 1 ( 8%) | 29 (30%) | 6 (60%) | 4 (31%) | 3 (60%) | 0 ( 0%) | 3 (27%) | <0.001 |
|  |  |  |  |  |  |  |  |  |  |
| Prior Trauma/Abd Surgery |  |  |  |  |  |  |  |  | <0.001 |
| No | 31 (91%) | 11 (92%) | 46 (48%) | 3 (30%) | 3 (23%) | 2 (40%) | 3 (43%) | 3 (27%) |  |
| Yes | 3 ( 9%) | 1 ( 8%) | 50 (52%) | 7 (70%) | 10 (77%) | 3 (60%) | 4 (57%) | 8 (73%) |  |
|  |  |  |  |  |  |  |  |  |  |
| Type of Injury |  |  |  |  |  |  |  |  | <0.001 |
| Penetrating | 34 (100%) | 7 (58%) | 0 ( 0%) | 0 ( 0%) | 0 ( 0%) | 0 ( 0%) | 0 ( 0%) | 0 ( 0%) |  |
| Blunt | 0 ( 0%) | 5 (42%) | 0 ( 0%) | 0 ( 0%) | 0 ( 0%) | 0 ( 0%) | 0 ( 0%) | 1 ( 9%) |  |
| Post-Surgical | 0 ( 0%) | 0 ( 0%) | 96 (100%) | 10 (100%) | 13 (100%) | 5 (100%) | 0 ( 0%) | 0 ( 0%) |  |
| None | 0 ( 0%) | 0 ( 0%) | 0 ( 0%) | 0 ( 0%) | 0 ( 0%) | 0 ( 0%) | 7 (100%) | 10 (91%) |  |
|  |  |  |  |  |  |  |  |  |  |
| Reason for ERCP |  |  |  |  |  |  |  |  | <0.001 |
| GSW | 34 (100%) | 0 ( 0%) | 0 ( 0%) | 0 ( 0%) | 0 ( 0%) | 0 ( 0%) | 0 ( 0%) | 0 ( 0%) |  |
| MVC | 0 ( 0%) | 12 (100%) | 0 ( 0%) | 0 ( 0%) | 0 ( 0%) | 0 ( 0%) | 0 ( 0%) | 0 ( 0%) |  |
| Cholecystectomy | 0 ( 0%) | 0 ( 0%) | 96 (100%) | 0 ( 0%) | 0 ( 0%) | 0 ( 0%) | 0 ( 0%) | 0 ( 0%) |  |
| Liver Transplant | 0 ( 0%) | 0 ( 0%) | 0 ( 0%) | 10 (100%) | 0 ( 0%) | 0 ( 0%) | 0 ( 0%) | 0 ( 0%) |  |
| Hepatectomy | 0 ( 0%) | 0 ( 0%) | 0 ( 0%) | 0 ( 0%) | 13 (100%) | 0 ( 0%) | 0 ( 0%) | 0 ( 0%) |  |
| Other Surgery | 0 ( 0%) | 0 ( 0%) | 0 ( 0%) | 0 ( 0%) | 0 ( 0%) | 5 (100%) | 0 ( 0%) | 0 ( 0%) |  |
| Pancreatitis | 0 ( 0%) | 0 ( 0%) | 0 ( 0%) | 0 ( 0%) | 0 ( 0%) | 0 ( 0%) | 7 (100%) | 0 ( 0%) |  |
| Other Medical Condition | 0 ( 0%) | 0 ( 0%) | 0 ( 0%) | 0 ( 0%) | 0 ( 0%) | 0 ( 0%) | 0 ( 0%) | 11 (100%) |  |
|  |  |  |  |  |  |  |  |  |  |
| Detection of Leak |  |  |  |  |  |  |  |  | <0.001 |
| CTAP | 23 (68%) | 6 (50%) | 30 (31%) | 5 (50%) | 6 (46%) | 1 (20%) | 2 (29%) | 4 (36%) |  |
| HIDA | 3 ( 9%) | 2 (17%) | 32 (33%) | 2 (20%) | 1 ( 8%) | 1 (20%) | 1 (14%) | 3 (27%) |  |
| MRCP | 2 ( 6%) | 1 ( 8%) | 3 ( 3%) | 0 ( 0%) | 1 ( 8%) | 0 ( 0%) | 0 ( 0%) | 1 ( 9%) |  |
| PCT/JP Biliary Drainage Increase | 5 (15%) | 2 (17%) | 19 (20%) | 1 (10%) | 4 (31%) | 0 ( 0%) | 0 ( 0%) | 0 ( 0%) |  |
| Intra-Op Visualization | 0 ( 0%) | 0 ( 0%) | 5 ( 5%) | 0 ( 0%) | 0 ( 0%) | 0 ( 0%) | 0 ( 0%) | 0 ( 0%) |  |
| US | 0 ( 0%) | 0 ( 0%) | 1 ( 1%) | 2 (20%) | 0 ( 0%) | 0 ( 0%) | 2 (29%) | 0 ( 0%) |  |
| Other | 1 ( 3%) | 1 ( 8%) | 6 ( 6%) | 0 ( 0%) | 1 ( 8%) | 3 (60%) | 2 (29%) | 3 (27%) |  |
|  |  |  |  |  |  |  |  |  |  |
| Stent Placed |  |  |  |  |  |  |  |  | 0.69 |
| No | 0 ( 0%) | 0 ( 0%) | 5 ( 5%) | 0 ( 0%) | 1 ( 8%) | 0 ( 0%) | 0 ( 0%) | 0 ( 0%) |  |
| Yes | 34 (100%) | 12 (100%) | 90 (95%) | 10 (100%) | 12 (92%) | 5 (100%) | 7 (100%) | 11 (100%) |  |
|  |  |  |  |  |  |  |  |  |  |
| Stent Exchange |  |  |  |  |  |  |  |  | 0.049 |
| No | 30 (88%) | 11 (92%) | 86 (91%) | 5 (50%) | 10 (77%) | 4 (80%) | 6 (86%) | 9 (82%) |  |
| Yes | 4 (12%) | 1 ( 8%) | 9 ( 9%) | 5 (50%) | 3 (23%) | 1 (20%) | 1 (14%) | 2 (18%) |  |
|  |  |  |  |  |  |  |  |  |  |
| Time to exchange [months] | 2.2 (1.0) | 4.0 (.) | 2.4 (1.2) | 0.8 (0.3) | 1.7 (0.6) | 1.0 (.) | 4.0 (.) | 1.0 (0.0) | 0.015 |
|  |  |  |  |  |  |  |  |  |  |
| Stent Removal |  |  |  |  |  |  |  |  | 0.5 |
| No | 9 (27%) | 5 (42%) | 18 (19%) | 3 (30%) | 5 (38%) | 1 (20%) | 3 (43%) | 3 (27%) |  |
| Yes | 24 (73%) | 7 (58%) | 77 (81%) | 7 (70%) | 8 (62%) | 4 (80%) | 4 (57%) | 8 (73%) |  |
|  |  |  |  |  |  |  |  |  |  |
| Time to stent removal [months] | 3.8 (1.6) | 2.9 (1.6) | 3.8 (2.8) | 2.7 (1.0) | 5.1 (3.2) | 2.2 (1.0) | 4.2 (3.9) | 2.6 (1.1) | 0.35 |
|  |  |  |  |  |  |  |  |  |  |
| Length of time of JP/PTC Drain placement | 3.3 (5.8) | 2.2 (2.9) | 2.5 (3.7) | 4.4 (6.6) | 1.2 (1.8) | 2.0 (.) | 1.0 (1.4) | 0.0 (0.0) | 0.78 |
|  |  |  |  |  |  |  |  |  |  |
| IR Involvement |  |  |  |  |  |  |  |  | 0.001 |
| No | 13 (38%) | 5 (42%) | 70 (73%) | 3 (30%) | 5 (38%) | 4 (80%) | 6 (86%) | 7 (64%) |  |
| Yes | 21 (62%) | 7 (58%) | 26 (27%) | 7 (70%) | 8 (62%) | 1 (20%) | 1 (14%) | 4 (36%) |  |
|  |  |  |  |  |  |  |  |  |  |
| IR Procedure |  |  |  |  |  |  |  |  | 0.087 |
| No | 3 (14%) | 1 (14%) | 9 (33%) | 2 (29%) | 5 (62%) | 1 (100%) | 1 (100%) | 2 (50%) |  |
| Yes | 18 (86%) | 6 (86%) | 18 (67%) | 5 (71%) | 3 (38%) | 0 ( 0%) | 0 ( 0%) | 2 (50%) |  |
|  |  |  |  |  |  |  |  |  |  |
| IR Drain Exchange? |  |  |  |  |  |  |  |  | 0.46 |
| No | 6 (27%) | 3 (50%) | 11 (46%) | 2 (29%) | 2 (25%) | 0 ( 0%) | 0 ( 0%) | 0 ( 0%) |  |
| Yes | 16 (73%) | 3 (50%) | 13 (54%) | 5 (71%) | 6 (75%) | 1 (100%) | 2 (100%) | 4 (100%) |  |
|  |  |  |  |  |  |  |  |  |  |
| LOS [days] | 22.7 (16.4) | 18.8 (14.1) | 8.9 (8.0) | 35.8 (33.0) | 12.2 (7.7) | 6.0 (6.1) | 12.6 (17.4) | 23.5 (37.3) | <0.001 |
|  |  |  |  |  |  |  |  |  |  |
| Grade |  |  |  |  |  |  |  |  | 0.024 |
| 0 | 1 ( 3%) | 0 ( 0%) | 13 (14%) | 1 (10%) | 1 ( 8%) | 0 ( 0%) | 0 ( 0%) | 1 ( 9%) |  |
| 1 | 16 (47%) | 4 (33%) | 16 (17%) | 1 (10%) | 2 (15%) | 0 ( 0%) | 1 (14%) | 0 ( 0%) |  |
| 2 | 17 (50%) | 8 (67%) | 67 (70%) | 8 (80%) | 10 (77%) | 5 (100%) | 6 (86%) | 10 (91%) |  |
|  |  |  |  |  |  |  |  |  |  |
| StrasbergClassification1 |  |  |  |  |  |  |  |  |  |
| A |  |  | 62 (65%) |  |  |  |  |  |  |
| D |  |  | 25 (26%) |  |  |  |  |  |  |
| E1 |  |  | 8 ( 8%) |  |  |  |  |  |  |
| E2 |  |  | 1 ( 1%) |  |  |  |  |  |  |
|  |  |  |  |  |  |  |  |  |  |
| Death |  |  |  |  |  |  |  |  | 0.085 |
| No | 34 (100%) | 12 (100%) | 92 (96%) | 8 (80%) | 13 (100%) | 5 (100%) | 6 (86%) | 11 (100%) |  |
| Yes | 0 ( 0%) | 0 ( 0%) | 4 ( 4%) | 2 (20%) | 0 ( 0%) | 0 ( 0%) | 1 (14%) | 0 ( 0%) |  |
|  |  |  |  |  |  |  |  |  |  |
| Complications |  |  |  |  |  |  |  |  | 0.2 |
| No | 32 (94%) | 12 (100%) | 93 (97%) | 8 (80%) | 13 (100%) | 5 (100%) | 7 (100%) | 11 (100%) |  |
| Yes | 2 ( 6%) | 0 ( 0%) | 3 ( 3%) | 2 (20%) | 0 ( 0%) | 0 ( 0%) | 0 ( 0%) | 0 ( 0%) |  |
|  |  |  |  |  |  |  |  |  |  |
| Type of complication |  |  |  |  |  |  |  |  | 0.35 |
| 1 (Abscess) | 0 ( 0%) |  | 1 (33%) | 0 ( 0%) |  |  |  |  |  |
| 1 (Biliary strictures) | 0 ( 0%) |  | 1 (33%) | 1 (50%) |  |  |  |  |  |
| 1 (Bleeding) | 1 (50%) |  | 0 ( 0%) | 0 ( 0%) |  |  |  |  |  |
| 1 (Hepatic infarction) | 1 (50%) |  | 0 ( 0%) | 0 ( 0%) |  |  |  |  |  |
| 1 (Pancreatitis) | 0 ( 0%) |  | 1 (33%) | 0 ( 0%) |  |  |  |  |  |
| Biloma | 0 ( 0%) |  | 0 ( 0%) | 1 (50%) |  |  |  |  |  |

Data are presented as mean (SD) for continuous measures, and n (%) for categorical measures.

Supplement 6. Lesions to other organs in traumatic patients.

|  | n | % |
| --- | --- | --- |
| Liver | 23 | 48.9% |
| Vascular | 4 | 8.5% |
| Hallow Viscus | 3 | 6.4% |
| Diafragm | 2 | 4.3% |
| Pancreas | 1 | 2.1% |
| Spleen | 4 | 8.5% |
| Lung | 15 | 31.9% |
| Cardiac | 0 | 0.0% |
| Other organ laceration | 5 |  |
